# Epistatic Control of Mammary Cancer Susceptibility in Mice may Depend on the Dietary Environment

**DOI:** 10.4172/2161-1041.1000108

**Published:** 2012-06-01

**Authors:** Larry J. Leamy, Ryan R. Gordon, Daniel Pomp

**Affiliations:** 1 Department of Biology, University of North Carolina at Charlotte, Charlotte, North Carolina, 28223; 2 Department of Nutrition, University of North Carolina, Chapel Hill, North Carolina 27599; 3 Department of Biology, Nutrition, and Cell and Molecular Physiology, University of North Carolina, Chapel Hill North Carolina, 27599

**Keywords:** Mammary cancer, PyMT, High-fat diet, Epistasis, QTLs, Mice

## Abstract

Recent studies have linked a high fat diet to the development of breast cancer, but any genetic basis for this association is poorly understood. We investigated this association with an epistatic analysis of seven cancer traits in a segregating population of mice with metastatic mammary cancer that were fed either a control or a high-fat diet. We used an interval mapping approach with single nucleotide polymorphisms to scan all 19 autosomes, and discovered a number of diet-independent epistatic interactions of quantitative trait loci (QTLs) affecting these traits. More importantly, we also discovered significant epistatic by diet interactions affecting some of the traits that suggested these epistatic effects varied depending on the dietary environment. An analysis of these interactions showed some were due to epistasis that occurred in mice fed only the control diet or only the high-fat diet whereas other interactions were generated by differential effects of epistasis in the two dietary environments. Some of the epistatic QTLs appeared to colocalize with cancer QTLs mapped in other mouse populations and with candidate genes identified from eQTLs previously mapped in this population, but others represented novel modifying loci affecting these cancer traits. It was concluded that these diet-dependent epistatic QTLs contribute to a genetic susceptibility of dietary effects on breast cancer, and their identification may eventually lead to a better understanding that will be needed for the design of more effective treatments for this disease.

## Introduction

Breast cancer is a particularly prevalent form of cancer that affects about 12% of all women at some point during their lifetime [1]. For 2010 in the United States, this translates into more than 200,000 women, with nearly 40,000 predicted to die [2]. Death typically results from secondary metastatic disease [3], with the five-year survival percentage being only 23.4% if the cancer has metastasized [1]. As a consequence, currently there is an intense focus on understanding the genetic and environmental factors controlling metastatic progression with an eventual goal of reducing the mortality associated with this disease.

Although mutations in genes such as *BRCA1* and *BRCA2* are well known to predispose individuals to an increased risk of breast cancer, it has become clear that the incidence of this disease primarily depends on the action of a number of polygenes [4–6]. Consistent with this, increasing numbers of quantitative trait loci (QTLs) that affect susceptibility to various mammary cancer traits have been detected, especially in mouse and rat models [7–9]. There is also mounting evidence that these QTLs often are not independent but instead epistatically interact with other loci to affect the incidence and development of this type of cancer [10–13]. Some investigators in fact have predicted that interactions of genes may prove to be more important than their single-locus effects in determining the susceptibility of individuals to breast cancer and other human diseases [14,15].

Beyond gene-gene interactions, interactions of genes with various environmental factors, especially diet, also are known to influence the risk of breast cancer [16–19]. Studies suggesting this have been conducted primarily with high-penetrance genes implicated in breast cancer, however, and comprehensive genome scans for QTL by diet interactions affecting cancer traits are almost non-existent. An exception is a recent study by Gordon et al. [9] who analyzed an F_2_ intercross population of mice fed either a control or a high fat diet and that carried the PyMT transgene causing them to develop mammary cancer. These investigators discovered QTLs on 10 different autosomes that affected one or more of 8 mammary cancer traits [9]. More importantly, the majority of these QTLs exhibited interactions with the dietary environment, suggesting loci for dietary response. Analysis of these loci showed that most of them were the result of QTL effects that were significant only in mice fed the high-fat and not the control diet [9].

Given these results indicating that single-locus effects of breast cancer QTLs can differ depending on the dietary environment, we were interested to know whether this might be true for two-locus (epistatic) effects as well. To test this, we made use of the same population of mice analyzed by Gordon et al. [9], and conducted a genome scan for epistasis and epistasis by diet interactions affecting the cancer traits. We wanted to determine the extent of epistasis affecting these traits and to see whether the cancer QTLs previously found by Gordon et al. [9] might be major players in the epistatic interactions discovered. Our primary interest, however, was to test for significant epistasis X diet interactions affecting the mammary cancer traits. If these exist, it would suggest that there is a genetic link between diet and breast cancer that extends beyond that due to single-locus effects of genes.

## Materials and Methods

### The population and traits

We used an F_2_ population of mice produced from an intercross of two inbred strains, M16i and FVB/NJ-TgN(MMTV-PyMT)^634Mul^ [9]. The M16i strain was derived from the M16 line that had been subjected to selection for 3- to 6 week body weight gain [20,21] and FVB contained a PyMT transgene that causes development of mammary tumors and subsequent pulmonary metastases [22]. At four weeks of age, the F_2_ mice were randomly allocated (within PyMT genotype and sex) into either a group fed a high-fat diet or a group fed a matched control fat diet. All mice were sacrificed at 11 (females) or 14 (males) weeks of age [23].

A total of 5 tumor and 3 metastatic traits were measured in all mice carrying the PyMT transgene. The tumor traits included the age of onset of tumors (TOID) recorded in days, as well as the total number of mammary tumors (TC) scored after sacrifice. In addition, weights (in g) were recorded for the inguinal (TI) and axillary (TA) tumors and their total (TTW = TI + TA). The metastatic traits were obtained by evaluating the metastases present on the lung surface and those observed in formalin-fixed lung sections. Specifically, these traits included the total number of surface metastases present (MET), and two traits measured only in female mice lung sections: the density of metastases (AMD), and the number of metastatic lesions per square micron of lung tissue (AMN). Further details regarding the scoring/measurement of these 8 traits are given in Gordon et al. [9].

For several reasons, the sample sizes for these 8 traits were reduced from the total number of PyMT+ mice (264: 106 males, 158 females), and also varied considerably among the traits. For the tumor traits, some mice did not develop tumors, and all zero values were treated as missing data because we could not determine whether these mice might have developed tumors if given more time beyond the sacrifice date. Thus the sample size for TOID was 210 and for TC and TA, it was 217. For TI the sample size was 149 because no inguinal tumors developed in males and thus this total was for females only. TTW also had a sample size of 149 since we used only the female values for the total of both inguinal and axillary tumors. The numbers of metastatic traits were reduced further because a substantial proportion of PyMT+ mice failed to develop the disseminated (lung) tumors by the time of sacrifice. Also, the values for MET in male mice all were 0 and thus we used female values only, their sample size being 63. For AMN and AMD, the total sample size (again for females only) was 86. For each trait, the number of mice fed the high-fat diet (mean = 55) was slightly higher than the number fed the control diet (mean = 51). In summary, phenotypic data of varying sample sizes were available for the analysis of all 8 traits in females, and TOID, TC, and TA in males.

We used tail samples to obtain DNA for single nucleotide polymorphism (SNP) genotyping of all F_2_ mice. A total of 124 fully informative SNPs that were polymorphic between the two strains were chosen to provide coverage of all 19 autosomes. A list of these SNP markers with their locations and intermarker distances in cM is provided in Gordon et al. [23]. We used all available information from this SNP genotyping in the development of the linkage map via Map Manager QTXb20 [24], and in the subsequent QTL analysis.

### Preliminary analyses

Prior to the epistasis analyses, we first examined the distributions of the eight tumor/metastatic traits (after adjustment for potential diet effects). All traits exhibited significant skewness, so we logged their values and this was successful in promoting normality in all traits except TC. In females, 124 of the total of 149 individuals exhibited 10 tumors, so the distribution of TC was not informative. Similarly, 57 of the 68 total males exhibited either 1 or 2 tumors; so for both sexes, we omitted this trait from the analysis. After the logarithmic transformation, we also tested for sexual dimorphism in the two traits measured in both sexes (TOID and TA), and results were significant (*P* < 0.01) in all cases. Preliminary epistasis analyses also showed that these two traits were affected by extensive sex by epistasis interactions. Because of these sex-specific effects, therefore, all epistastic analyses of TOID and TA were conducted separately for males and for females. Epistastic analyses of the 5 remaining traits were conducted only for females.

To implement the epistatic analyses, we initially derived additive and dominance genotypic index values at and between flanking SNP markers on each chromosome in the manner previously described [25]. This allowed us to conduct scans for epistasis and epistatic interactions affecting the tumor and metastatic traits among all pairs of locations 2 cM apart for each of the 171 pairs of 19 autosomes.

### Diet-independent epistasis scans

For the diet-independent epistasis scans, we used the MIXED procedure in SAS (SAS, version 9.2; SAS Institute, Cary, NC) with the following model:

(1) $$\begin{array}{l} \text{y}=\mu +\text{dam}+\text{litters}+\text{diet}+(\text{age})+a1{\text{X}}_{a1}+d1{\text{X}}_{d1}+a2{\text{X}}_{a2}+d2{\text{X}}_{d2}\\ +aa({{\text{X}}_{a1}}^{★}{\text{X}}_{a2})+ad({{\text{X}}_{a1}}^{★}{\text{X}}_{d2})+da({{\text{X}}_{d1}}^{★}{\text{X}}_{a2})+dd({{\text{X}}_{d1}}^{★}{\text{X}}_{d2})+\varepsilon \end{array}$$

In this model y is the trait, and the independent variables included are the additive (X*_a1_* and X*_a2_*) and dominance genotypic index values (X*_d1_* and X*_d2_*), the X*_a1_*^★^X*_a2_*, X*_a1_*^★^X*_d2_*, X*_d1_*^★^X*_a2_*, and X*_d1_*^★^X*_d2_* terms that represent their pairwise epistatic products, and the residual, ε. The model included two random classification factors, replicate litters and dams [23], and diet as a fixed classification factor used to adjust for potential effects of differences in the control versus high fat diet. Age also was used as a covariate for those traits measured at sacrifice (all except TOID) to adjust for any effects due to age differences.

These analyses generated a −2 ln likelihood value at each pair of sites that we subtracted from the comparable value at the same sites obtained in a reduced model that included only the dam, litters, and diet (and where appropriate, age) terms. We evaluated the difference between these two values via a chi-square test with 8 degrees of freedom, and transformed its associated probability into a LOD score as follows: log_10_(1/probability). If the highest LOD score for each combination of chromosomes exceeded the appropriate 5% genomewise threshold value (see below), it was considered significant. For those pairs of sites reaching significance in the full model, we subtracted the 2 ln likelihood value generated from the comparable value obtained in a reduced model containing all except the four epistasis terms. This difference was evaluated with a chi-square test associated with 4 degrees of freedom, and if significant (*P* < 0.05), epistasis was assumed to be present. All combinations exhibiting diet-independent epistasis also were tested for interactions with diet (see below), although none reached significance in these tests.

Where significant diet-independent epistasis occurred, we estimated the four orthogonal epistatic components (*aa, ad, da, dd*) from regression coefficients and tested them for significance (*P* < 0.05) using single degree-of-freedom *F*-tests. The *aa* (additive by additive) mode of epistasis occurs when the single-locus additive genotypic value (half the difference of the values for the two homozygotes) at a given locus (A) differs depending on what genotype (*B/B, B/b,* or *b/b*) is at another locus (B) and *vice versa*. The *ad* (additive by dominance) mode of epistasis occurs when the single locus additive genotypic value for a locus A differs depending on the genotype at another locus B whereas the single-locus dominance genotypic value (difference between the heterozygous and mid-homozygote values) at B differs depending on the locus A genotype (and vice versa for *da* epistasis). Finally, the *dd* (dominance by dominance) mode of epistasis occurs when the single-locus dominance genotypic value at locus A differs depending on the genotype at locus B and vice versa [26].

It also was of interest to calculate the impact of diet-independent epistasis on the phenotypic variation in each of the cancer traits. We did this by first adjusting each trait for the effects of dams, litters, and diet (and where appropriate, age) with the reduced mixed model described above. For each trait, we then ran two separate multiple regression analyses on the residuals generated from the mixed model. In one model, the independent variables were all single-locus effects associated with each two-locus epistatic interaction that reached significance in the epistasis runs; in the second model, the independent variables included both these single locus variables as well as all epistatic components (*aa*, etc.) reaching significance. The percentage effect of epistasis was then assessed as (100 times) the difference between the adjusted R^2^ values from the two models. Thus for each trait, this value assessed the variation contributed by epistasis beyond that contributed by single-locus effects.

### Diet-dependent epistasis scans

We also performed similar genome scans for each of the traits to search for interactions of epistasis with diet (diet-dependent epistasis). These scans used a basic model similar to (1) above but which included four interactions of the pairwise epistatic products with diet:

(2) $$\begin{array}{l} \text{y}=\mu +\text{dam}+\text{litters}+\text{diet}+(\text{age})+a1{\text{X}}_{a1}+d1{\text{X}}_{d1}+a2{\text{X}}_{a2}+d2{\text{X}}_{d2}\\ +aa({{\text{X}}_{a1}}^{★}{\text{X}}_{a2})+ad({{\text{X}}_{a1}}^{★}{\text{X}}_{d2})+da({{\text{X}}_{d1}}^{★}{\text{X}}_{a2})+dd({{\text{X}}_{d1}}^{★}{\text{X}}_{d2})\\ +\mathrm{aad}({{\text{X}}_{a1}}^{★}{{\text{X}}_{a2}}^{★}\text{diet})+\mathrm{add}({{\text{X}}_{\text{a}1}}^{★}\text{Xd}2^{★}\text{diet})+\mathrm{dad}({{\text{X}}_{d1}}^{★}{{\text{X}}_{a2}}^{★}\text{diet})+\\ \mathrm{ddd}({{\text{X}}_{\text{d}1}}^{★}{{\text{X}}_{\text{d}2}}^{★}\text{diet})+\varepsilon \end{array}$$

This full model was tested for significance by comparing its −2 ln likelihood value again with that from a reduced model using only dam, litters, and diet (also age where appropriate). The difference between these likelihood values from these two models was evaluated by a chi-square statistic associated with 12 d.f., and was considered significant if the LOD score calculated from the probability exceeded the threshold level of significance. Where significance occurred, epistasis by diet interactions were tested by comparing the full model (2) above with another reduced model identical to the full model but not containing the four epistasis by diet interaction terms. Those (4 d.f.) chi-square tests reaching significance (*P* < 0.05) were considered to indicate significant diet by epistasis interactions affecting the traits.

When significant diet by epistasis interactions occurred, they were interpreted as representing epistasis for dietary response. We investigated the nature of this response by testing for significant epistasis separately in each of the two (control and high fat) diet groups. We used model (1) above and the same approach already described to calculate LOD scores in each dietary group. LOD scores of 1.30 (*P* < 0.05) or higher were regarded as statistically significant. Using this criterion, we tallied the number of epistatic effects for each trait that were significant in one dietary environment but not the other, as well as the remaining number of those showing significant LOD scores in both groups and non-significant LOD scores in both groups (differential epistatic effects).

### Threshold levels of significance

We conducted hundreds of epistasis and epistasis by diet interaction tests for each of the seven cancer traits, and it therefore was important to establish appropriate threshold levels of significance. We proceeded by first calculating the number of independent markers on each chromosome using the method advocated by Li and Ji [27]. The sum of the crossproducts of these values was 3172, which is an estimate of the total number of independent epistasis tests. This enabled us to calculate a 0.05 Bonferroni threshold probability (*P*) by 0.05/3172 = 0.0000157, equivalent to a LOD score of log_10_ (1/*P*) = 4.80. According to this calculation, any test for epistasis or an epistasis X diet interaction generating a LOD score of 4.80 or higher would be considered significant at the 5% genomewise level. We also derived a threshold for suggestive epistasis (and epistasis X diet interactions) by dividing 0.2 by 3172 that translates into a LOD score of 4.20. This seemed an appropriate compromise between the stringent 5% genomewise level and the quite liberal definition of suggestive linkage proposed for single-locus testing by Lander and Krugylak [28] as a LOD score that is expected to occur in a genome scan once by chance alone (1/3172; LOD = 3.50). We found the Lander/Kruglyak threshold useful to compare trends among the epistatic interactions (see the discussion below), and refer to all interactions associated with a LOD score of 3.50 or better as meeting the liberal threshold. However, results are presented only for those epistatic interactions reaching the suggestive or significant thresholds.

The validity of the suggestive and significant threshold LOD scores estimated as described above depends on whether the epistasis models conform to the null model expectation. Thus with no epistatic effects, these models should generate a LOD score of 1.30 (equivalent to a probability of 0.05) 5% of the time and a LOD of 0.70 20% of the time. To test this for each trait, we simulated 1000 samples (with size equal to that for the specific trait) of two independent loci (A and B) each with two alleles (A_1_ and A_2_, B_1_ and B_2_) at equal frequencies. We assigned additive genotypic index values of −1, 0, and +1 and dominance genotypic index values of −0.5, +0.5, and 0.5, respectively, for A_1_A_1_ and B_1_B_1_ homozygotes, A_1_A_2_ and B_1_B_2_ heterozygotes, and A_2_A_2_ and B_2_B_2_ homozygotes. For each trait, we then ran the epistasis and epistasis X diet models as before but using the simulated genotypic data to generate LOD scores. This allowed us to examine the 95th and 80th percentile values in the LOD score distributions to see if they conformed to expectations. In nearly all instances, we found that the LOD scores exceeded the expected values, suggesting that our models for most traits were biased and that the thresholds of 4.80 and 4.20 were too low.

To arrive at more appropriate thresholds, therefore, we generated simulations as before, but this time for each of 3, 6, 12, 20, and 50 independent pairs of loci. We ran the epistasis models for each trait on 1000 simulated samples and identified the highest LOD score in each run. We then used the 95th (and 80th) percentile value from the LOD distributions from each of the five cases of different numbers of loci for use as the dependent variable in a linear regression. In this model, the log of the number of pairs of loci (n) served as the independent variable, allowing us to estimate the y-intercept *a*, and the slope, *b*: LOD = *a* + *b*[log_10_(n)]. This equation shows that the LOD scores should increase linearly with increasing numbers of locus pairs. For each trait, we found this to be the case, and therefore used the intercepts and slopes calculated from these regressions to predict the threshold value for n = 3172 tests. In this manner, we estimated significant and suggestive threshold LOD values specific for each of the traits in tests both of diet-independent and diet-dependent epistasis. All estimated significant thresholds in fact exceeded 4.80, and 13 of the 14 suggestive thresholds exceeded 4.20 (see Results below).

## Results

**Figure 1:**
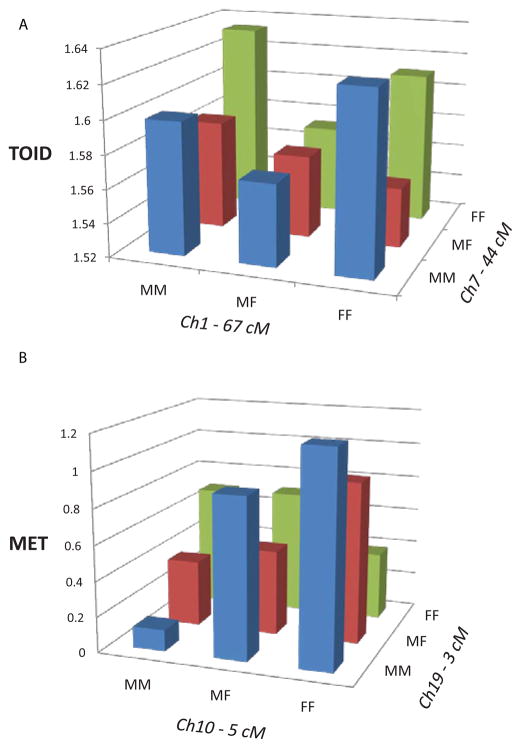
Epistatic interactions between QTLs on chromosomes 1 and 7 affecting TOID in female mice (A) and between QTLs on chromosomes 10 and 19 affecting MET in female mice (B). MM = M16i/M16i homozygotes, MF = M16i/FVB heterozygotes, and FF = FVB/FBV homozygotes.

We discovered a total of 28 diet-independent epistatic interactions affecting the cancer traits (Table 1). Only two of these interactions generated LOD scores that exceeded the 5% genomewise threshold values, one involving QTLs on chromosome 4 and 17 affecting TOID in males, and a second involving QTLs on chromosomes 1 and 3 affecting AMN in females. All other interactions were suggestive of epistasis. Nearly 1/3 of all interactions (9) occurred for TOID in males, although no epistasis was detected for TA in males. Two epistatic interactions affected TOID in females, neither of which is the same for those affecting TOID in males. Some interactions affected more than one trait, but these instances of epistatic pleiotropy occurred mostly for closely associated traits. Thus both epistatic interactions affecting AMN are precisely the same as two of the three affecting AMD. Similarly, QTLs involved in three of the five interactions affecting TI are at the same or similar locations on identical chromosomal pairs as three of the four affecting TTW (TI + TA). Chromosome 1 was most involved in these interactions (9 occurrences), and was particularly noticeable for AMN and AMD where it occurred in all five interactions affecting these two traits. Significant effects for the *aa* and *dd* epistatic values appeared to predominate among the tumor and (especially) the metastatic traits, but the overall distribution of *aa, ad*/*da*, and *dd* values (17, 27, 19) followed the expected 1:2:1 ratio (*P* = 0.49 in a chi-square test). The percentage impact of the significant epistatic components on the phenotypic variability in the traits was substantial, ranging from 15% to 41% and averaging 27.1%.

(Figure 1A) illustrates an example of primarily dominance by dominance (*dd*) epistatic effects on TOID in females generated by an interaction of QTLs on chromosomes 1 and 7. We calculated the expected values for each of the nine genotypes in this interaction from the coefficients produced in the epistasis solutions and the expectations given by Wolf et al. [29]. Note that the chromosome 1 QTL showed underdominance (heterozygote less than either homozygote) when associated with the homozygous genotypes on chromosome 7, but partial dominance when associated with the heterozygous genotype on chromosome 7. Similarly, the chromosome 7 QTL exhibited underdominance, but only when associated with the homozygous genotypes of the chromosome 1 QTL. (Figure 1B) illustrates a more complicated pattern of significant additive by additive and dominance by dominance effects on MET in females generated from a pair of QTLs on chromosome 10 and 19. Note that the additive effect (difference between the homozygotes) of the QTL on chromosome 10 was particularly pronounced when associated with the MM chromosome 19 homozygote. Additionally, the QTL on chromosome 19 exhibited partial dominance when associated with the homozygous genotypes of the chromosome 10 QTL, but underdominance when associated with the chromosome 10 heterozygous genotype.

**Figure 2:**
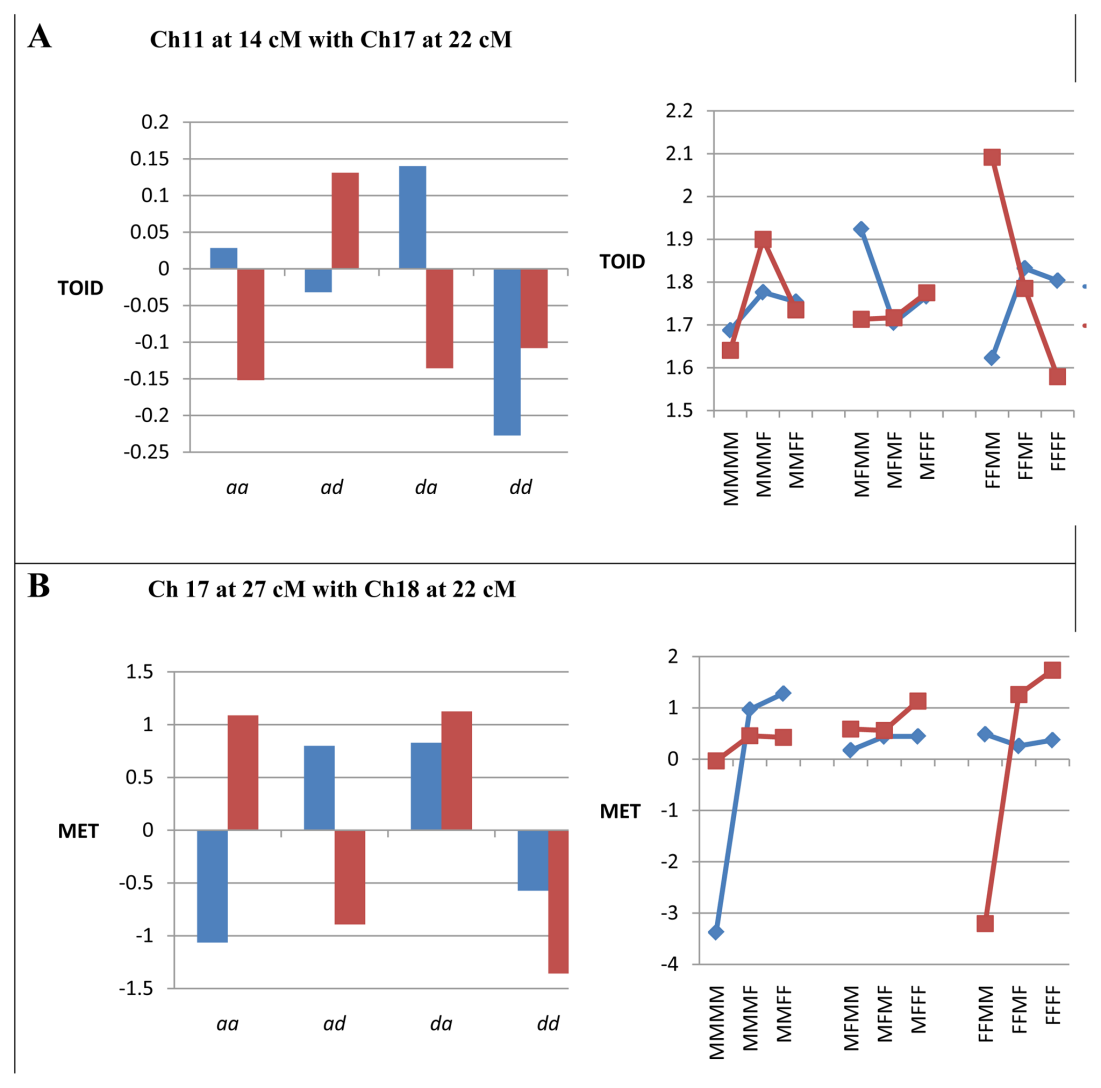
Epistatic effects on cancer traits in mice fed separate diets. Each example includes a bar diagram that shows the epistatic effects of two QTLs on the trait in each dietary group (* = *P* < 0.05). There also is a second line diagram that shows how epistasis in each group affects the relationship among the nine genotypic values (M = gene from M16i line, F = gene from FVB line) generated from the interaction of the two QTLs. Figure 2A illustrates epistatic effects of QTLs on chromosomes 11 and 17 on TOID in male mice that are significant in both dietary environments (differential effects). Figure 2B illustrates differential epistatic effects of QTLs on chromosomes 17 and 18 on MET in female mice.

We discovered a total of 22 diet by epistasis interactions affecting the cancer traits (Table 2). The genomewise threshold LOD scores tended to be higher than those for the epistasis analyses (Table 1), although two diet by epistasis interactions reached significance at this level. Both occurred in male mice, one affecting TOID and one affecting TA. More than half of these interactions (13) affected TOID in males, with none being detected for TA, TTW, and AMN in females. As was the case with the epistasis results, the chromosome pairs involved in the diet by epistasis interactions affecting TOID in males were quite distinct from those affecting this trait in females. Chromosomal pairs for all 22 interactions were distinct with chromosomes 1 and 11 involved in the greatest number of interactions (5 each). The distribution of significant interactions of the *aa, ad/da,* and *dd* components with diet over all traits (13, 23, and 14) followed the expected 1:2:1 ratio (*P* = 0.84 in a chi-square test). For nine of the epistasis by diet interactions, significant epistasis occurred about equally in mice fed the control versus the high fat diet (4:5). For the remaining 13 interactions, differential effects occurred in which there was significant epistasis in both dietary environments or in neither dietary environment.

(Figure 2) illustrates two examples of epistatic effects that differ in the two dietary environments. (Figure 2A) illustrates epistatic effects on TOID in males generated from the interaction of QTLs on chromosomes 11 and 17. Notice that the *aa, ad,* and *da* epistatic effects differed in sign (differential epistasis) in male mice fed the control versus the high fat diet. In the line diagram, this effect may be seen by the varying degrees of dominance especially noticeable in the high fat diet. (Figure 2B) shows the effects of epistasis on MET in female mice generated from an interaction of QTLs on chromosome 17 and 18. Again differential epistasis was present whereby the *aa* and *ad* epistatic components differ in sign in mice fed the control versus the high fat diet. Particularly noticeable were the extreme values for the MMMM control females and the FFMM females fed the high fat diet.

## Discussion

### Diet-independent epistatic effects

The primary purpose of this study was to test for epistasis and epistasis X diet interactions affecting the mammary cancer traits in our population of mice. We expected to detect epistasis because significant epistatic interactions of genes affecting these or similar traits have been reported in several studies with mice and rats [7,10,12,13,30,]. In fact we discovered a total of 28 diet-independent epistatic interactions, one or more of which affected all of the cancer traits except TA in males. While only two of the 28 interactions were significant at the 5% experimentwise level, the remaining 26 suggestive instances also offer strong evidence for diet-independent epistasis. This is because we established a rather stringent suggestive threshold level ensuring that in the entire genome scan for each trait, there should have been only a 20% chance of a false positive. Since we did analyze seven traits, however, this does imply that one (0.2 × 7 = 1.4) or perhaps two of the 26 suggestive interactions might be false instances of diet-independent epistasis.

It is of interest to determine the extent to which the diet-independent epistatic interactions might involve QTLs for the cancer traits mapped by Gordon et al. [9]. We compared single and two-locus QTL locations, and found that 24 of the 56 total QTLs from the 28 epistatic pairs (Table 1) were located within 20 cM of one or more of the cancer QTLs. This result suggests that less than one half of the QTLs epistatically affecting the cancer traits may be the same as the cancer QTLs previously identified in this population. The remaining epistatic QTLs are new to this mouse population, and represent additional modifying loci affecting the cancer traits. The two diet-independent epistatic interactions reaching genomewise significance provide examples. One of these interactions involves QTLs on chromosomes 4 (72 cM) and 17 (19 cM) affecting TOID in males (Table 1). Gordon et al. [9] detected a QTL on chromosome 17 affecting TOID in males (although at 51 cM), but found no QTLs on chromosome 4. We also discovered an epistatic interaction involving QTLs on chromosomes 1 (36 cM) and 3 (46 cM) that significantly affected MET in females. Although the chromosome 1 epistatic QTL may be the same as a QTL previously found on this same chromosome (at 7 cM), Gordon et al. [9] did not find any cancer QTLs on chromosome 3.

Some of the diet-independent epistatic QTLs may be the same as cancer loci previously mapped in other mouse populations. This seems particularly likely for epistatic QTLs on chromosomes 9 and 19. Thus we found a QTL on chromosome 9 (at 54–56 cM) epistatically interacting with QTLs on chromosomes 8 and 13 to affect TOID in males, and it colocalizes with a QTL on this chromosome at 55 cM previously found to affect tumor latency (*Apmt2*; [10]) and tumor number (*Mmom1*;[8]). Similarly, the QTL we found on chromosome 19 (4–9 cM) interacting with QTLs on chromosomes 10 and 11 to affect MET in females may very well be the same as a QTL (*Mtes1*) previously mapped on this chromosome (at 4 cM) that also affects metastasis of mammary cancer [31]. With the liberal threshold, two additional epistatic interactions of the QTL on chromosome 19 (at 5 cM) with QTLs on chromosome 14 and 18 affect MET. These results suggest that this chromosome 19 QTL may be an important player in diet-independent epistatic interactions that influence metastasis of mammary cancer.

The diet-independent epistatic interactions we discovered featured a rich architectural mixture, with the significant orthogonal components (*aa, ad/da,* and *dd*) present in expected (1:2:1) proportions and contributing substantially (average of 27%) to the phenotypic variation in the cancer traits. In general those traits affected by the greatest number of significant components (especially TOID in males) showed the highest percentage contributions, although just two significant components from a single epistatic interaction accounted for 15.2% of the variation in TI in males (Table 1). Given the small sample sizes involved for the cancer traits as well as the possibility of one or two false instances of epistasis, however, these contributions likely are inflated to some degree. On the other hand, we did not use any of the diet-dependent epistatic interactions in our calculations, and their inclusion surely would have increased the percentage estimates. So although we cannot know what the precise impact of epistasis was on the cancer traits, it appears to be considerable.

### Diet-dependent epistatic effects

While it was instructive to document the numbers and patterns of diet-independent epistatic effects on the cancer traits, our major objective was to see whether we could discover effects from epistatic interactions that could be altered by the dietary environment (diet-dependent epistasis). In fact we found 22 epistasis by diet interactions affecting the cancer traits, including two that were significant at the 5% genomewise level. This level is somewhat less than the 28 instances of epistasis alone affecting the traits, although the significant and suggestive threshold LOD values (means = 5.89, 4.90) used for testing these interactions were somewhat higher than those used in the epistasis tests (means = 5.52, 4.54). Use of the liberal threshold generated 90 diet-dependent epistatic interactions affecting the cancer traits (results not shown), nearly the same as the 91 instances of diet-independent epistatic interactions. In general, therefore, we have evidence that, like single-locus effects of QTLs [9], epistatic effects on the cancer traits can be influenced by the dietary environment.

Although chromosomal contributions in the diet-independent and diet-dependent epistatic interactions (Tables 1 and 2) appear to be generally similar, for any given trait, the chromosomes and the QTLs they harbor may differ considerably between these two types of interactions. For example, MET was affected by only one diet-dependent epistatic interaction (Table 2; Figure 2), and it did not involve the QTL on chromosome 19 (at 4–9 cM) that was so prevalent among diet-independent interactions affecting this trait (Table 1). Chromosome 19 is involved in three of the 16 diet by epistasis interactions generated using the liberal threshold, but the QTLs in two of these three instances may not be the same because they are more distally located (19 and 33 cM). A QTL on chromosome 17 (at 27–29 cM) is much more prominent, however, being involved in 6 of the 16 interactions. This QTL may be the same as one affecting mammary tumor metastatic progression found by Hunter et al. (2001) at a nearly identical position (25 cM) on chromosome 17. Whatever its identity, however, this QTL appears to play a central role in epistatic interactions affecting MET. But unlike the chromosome 19 QTL, the epistatic effects of this QTL on chromosome 17 depend entirely on the dietary environment. In fact, no QTL on chromosome 17 was involved in any of the 13 diet-independent epistatic interactions affecting MET generated with the liberal threshold. We should expect that QTLs with these sorts of diet-dependent effects often will be missed in standard epistasis analyses conducted with homogeneous dietary environments or in those in which adjustments are made for potential dietary effects.

At the sites on the 22 pairs of chromosomes where diet-dependent epistatic interactions affected the cancer traits (Table 2), significant epistasis occurred about equally in mice fed the control (4) and high fat diet (5). For the 90 epistasis by diet interactions produced using the liberal threshold, however, significant epistasis occurred more often in male mice fed the high fat rather than the control diet (9:2; *P* = 0.034 in a chi-square test) whereas the reverse was true for females (7:20; *P* = 0.012 in a chi square test). In females this trend was particularly apparent for TOID where 11 significant diet by epistasis interactions occurred in the control group and none occurred in the high fat group. This trend for females is opposite to that found (in both sexes) by Gordon et al. [9] in their single-locus QTL analysis of the cancer traits. This suggests that the expression of single- and two-locus effects of QTLs on these traits can vary considerably depending on the dietary environment.

As an example of this, Gordon et al. [9], found a QTL on chromosome 13 (19 cM) that affected TOID in females equally in both the high-fat and control diet. But we also found a QTL on chromosome 13 (at 13 cM) that interacted with a QTL on chromosome 5 (at 29 cM) to affect TOID significantly only in mice fed the control diet (Table 2). These QTLs on chromosome 13 may well represent the same gene, and if so, this suggests that the single-locus effects of this gene are altered by another (epistatic) gene, but only in mice fed the control diet. Other QTLs [9] and epistatic interactions of QTLs (Table 2) may affect TOID in females as well, so the net genetic impact on this trait would be sum of all of these effects. Only if the preponderance of all such effects on cancer traits tends to be expressed in one (e.g., high fat) dietary environment might we expect to find a genetic link between diet and mammary cancer.

### Identity of the QTLs

What might be the identity of these epistatic QTLs affecting the cancer traits? Fortunately, some possibilities may be inferred from an eQTL analyses conducted by Gordon et al. [32] and La Merrill et al. [33] using female PyMT mice from this same population. Gordon et al. [32] found over 200 *cis*-acting eQTLs scattered among all 19 autosomes, with a number being possible candidate genes for the diet-independent QTLs we discovered. Two examples are *Man2a1* that is a candidate for an epistatic QTL on chromosome 17 affecting MET, and *Wwc2* that is a candidate for an epistatic QTL on chromosome 8 affecting TI and AMD. Gordon et al. [32] also tested for interactions of *cis* eQTLs with diet, and detected just 15 significant instances of these interactions, the majority (9) of which were generated by significant eQTL effects in the control diet only. Among these 15 eQTLs, *Gdi3, D14Ertd449e,* and *Notch4*, respectively, appear to be reasonable candidates for diet-dependent epistatic QTLs on chromosomes 13, 14, and 17. La Merrill et al. [33] developed a list of candidate metastasis virulence and found that a central player in this network was *Vegfa*, vascular endothelial growth factor A. This gene was upregulated in mice fed the high-fat diet [33], and has been associated with metastatic breast cancer [34]. *Vegfa* is located on chromosome 17 at 23 cM, and thus is a candidate for the epistatic QTL we discovered on this chromosome (at 27 cM) interacting with a QTL on chromosome 18 to affect MET (Table 2).

## Conclusions

We have demonstrated that epistasis affects the cancer traits measured in our F_2_ population of mice. While this was not unexpected because of occasional reports of significant interactions of cancer QTLs, our genome scan revealed a number of novel QTLs throughout the genome that participate in interactions affecting these kinds of traits. More importantly, however, we also have shown that these epistatic effects sometimes vary depending on the dietary environment. Although Gordon et al. [9] showed that single-locus QTL effects on these traits are often diet-dependent, we now can say that this appears to be the case for two-locus, epistatic effects of QTLs as well. The link between diet and the development of cancer remains obscure, but clearly its genetical basis must involve some of the diet-dependent two-locus epistatic interactions we have uncovered. Identifying the candidate genes that these QTLs represent will be difficult, but once this is accomplished and the role of some of the major players elucidated, this should help us better understand metastatic cancer in individuals and design more effective personalized treatments and interventions for this disease.

## Figures and Tables

**Table 1:** Summary of epistatic interactions affecting the tumor and metastatic traits in male and female mice. Shown are the locations (Loc1 and Loc2) on each pair of chromosomes (Ch1 and Ch2) of the individual diet-independent epistatic interactions affecting the tumor and metastatic traits that reached significance at the 0.05 (bolded values) or the 0.2 (suggestive) genomewise levels. Significant and suggestive threshold LOD scores are given in parentheses in the headings for each trait. Epistatic components (*aa, ad, da, dd*) that reached significance at *P* <0.05 are indicated by asterisks and their cumulative percentage (%) contribution to the phenotypic variance of each trait are given.

Trait	Ch1	Ch2	Loc1	Loc2	LOD	*aa*	*ad*	*da*	*dd*	%
MALES										
TOID (5.768, 4.474)										41.3
TOID	1	10	21	31	4.49		*	*	*	
TOID	1	11	33	42	4.85	*			*	
TOID	2	11	18	72	4.51	*	*	*		
TOID	4	11	9	40	4.58	*	*	*	*	
TOID	4	15	72	10	4.50		*	*	*	
TOID	4	17	72	19	5.85		*	*	*	
TOID	8	9	19	56	4.64	*	*			
TOID	9	13	54	33	5.16		*	*	*	
TOID	10	11	31	44	4.59	*		*	*	
TA (5.855,4.881)										
FEMALES										
TOID (5.719, 4.986)										17.0
TOID	1	7	67	44	5.29				*	
TOID	10	17	66	46	5.07	*		*	*	
TA (5.307, 4.319)										32.2
TA	1	19	54	51	4.38	*	*			
TA	2	14	72	56	4.75	*	*		*	
TA	3	12	74	7	4.57	*		*	*	
TA	12	13	7	22	4.54	*	*			
TA	14	17	35	37	4.51			*	*	
TI (5.056, 4.220)										15.2
TI	2	4	91	70	4.50	*	*			
TTW (5.133, 4.050)										32.1
TTW	3	12	74	7	4.60	*		*	*	
TTW	5	11	79	5	4.29				*	
TTW	12	13	7	21	4.55	*	*			
TTW	14	17	35	37	4.09			*	*	
MET (6.081, 4.962)										28.7
MET	10	19	5	3	5.49	*			*	
MET	11	19	16	9	4.97			*	*	
AMN (5.202, 4.429)										26.2
**AMN**	**1**	**3**	**36**	**46**	**5.35**	*	*			
AMN	1	7	47	16	4.89				*	
AMN	1	8	47	12	4.47	*				
AMD (5.542, 4.511)										24.0
AMD	1	3	56	46	5.14	*	*			
AMD	1	7	50	47	4.91				*	

**Table 2:** Summary of the diet by epistasis interactions affecting cancer traits in male and female mice. Shown are the locations (Loc1 and Loc2) on each pair of chromosomes (Ch1 and Ch2) of the individual diet-dependent epistatic interactions affecting the tumor and metastatic traits that reached significance at the significant 0.05 (bolded values) or at the 0.2 (suggestive) genomewise level. Significant and suggestive threshold LOD scores are given in parentheses in the headings for each trait. Interactions of the epistatic components with diet (*aad, add, dad, ddd*) that reached significance at *P <* 0.05 are indicated by asterisks. For each interaction, LOD scores from tests of epistasis in mice fed the control (LOD-C) and high-fat diet (LOD-H) also are given.

Trait	Ch1	Ch2	Loc1	Loc2	LOD	*aad*	*add*	*dad*	*ddd*	LOD-C	LOC-H
MALES											
TOID (6.173, 5.025)											
TOID	1	2	23	97	5.77	*	*		*	1.00	0.76
TOID	1	3	23	44	6.16		*			.	1.38
TOID	1	9	16	38	5.77	*	*		*	0.91	0.78
TOID	1	17	24	25	5.29			*	*	3.70	0.00
TOID	2	4	42	72	5.27		*		*	5.89	2.90
TOID	2	16	36	26	5.11	*	*	*	*	0.03	2.92
TOID	4	11	66	55	5.79	*	*	*	*	3.57	2.55
TOID	8	11	31	3	5.79				*	1.09	5.32
TOID	8	12	53	31	5.42	*		*		3.23	3.38
TOID	10	11	31	33	5.38			*	*	2.95	3.76
TOID	11	12	44	37	5.08	*		*	*	2.32	6.17
**TOID**	**11**	**17**	**16**	**33**	**6.21**	*		*		**3.25**	**8.86**
TOID	16	17	32	23	5.97	*		*	*	0.09	2.82
TA (6.367, 5.370)											
TA	2	9	97	14	5.63		*	*		7.68	3.43
**TA**	**13**	**15**	**5**	**52**	**6.37**	*	*		*	**0.33**	**4.42**
FEMALES											
TOID (5.783, 4.837)											
TOID	1	14	67	17	5.20		*	*		4.07	0.42
TOID	5	13	29	13	5.72		*		*	4.02	0.51
TOID	5	18	28	31	5.10				*	2.62	0.28
TA (5.691, 4.666)											
TI (5.254, 4.177)											
TI	3	12	74	9	4.24	*		*		2.26	3.03
TI	8	13	29	59	5.07	*		*	*	3.78	1.62
TTW (5.675, 4.545)											
MET (6.781, 5.807)											
MET	17	18	27	22	5.97	*	*			2.59	1.74
AMN (5.625, 4.943)											
AMD (5.637, 4.688)											
AMD	8	14	30	23	4.69	*				1.26	2.33

## Abbreviations

QTLs: Quantitative trait loci
SNP: Single nucleotide polymorphism
LOD: Logarithm of the odds

## References

[1] Howlader N, Noone AM, Krapcho M, Neyman N, Aminou R (2008). SEER Cancer Statistics Review.

[2] American Cancer Society (ACS) Cancer Facts & Figures- 2010.

[3] Sporn MB (1996). The war on cancer. Lancet.

[4] Pharoah PDP, Antoniou A, Bobrow M, Zimmern RL, Easton DF (2002). Polygenic susceptibility to breast cancer and implications for prevention. Nat Genet.

[5] Peto J (2002). Breast cancer susceptibility—A new look at an old model. Cancer Cell.

[6] Balmain A, Gray J, Ponder B (2003). The genetics and genomics of cancer. Nat Genet.

[7] Samuelson DJ, Aperavich BA, Haag JD, Gould MN (2005). Fine mapping reveals multiple loci and a possible epistatic interaction within the mammary carcinoma susceptibility quantitative trait locus, *Mcs5*. Cancer Res.

[8] Blackburn AC, Hill LZ, Roberts AL, Wang J, Aud D (2007). Genetic mapping in mice identifies DMBT1 as a candidate modifier of mammary tumors and breast cancer risk. Am J Pathol.

[9] Gordon R, Hunter K, La Merrill M, Sørensen P, Threadgill DW (2008). Genotype X diet interactions in mice predisposed to mammary cancer: II. Tumors and metastases. Mamm Genome.

[10] Le Voyer T, Lu Z, Babb J, Lifsted T, Williams M (2000). An epistatic interaction controls the latency of a transgene-inducted mammary tumor. Mamm Genome.

[11] Ritchie MD, Hahn LW, Roodi N, Bailey LR, Dupont WD (2001). Multifactor-dimensionality reduction reveals high-order interactions among estrogen-metabolism genes in sporadic breast cancer. Am J Hum Genet.

[12] Wang H, Teske D, Tess A, Kohlhepp R, Choi Y (2007). Identification of novel modifier loci of *ApcMin* affecting mammary tumor development. Cancer Res.

[13] Piessevaux G, Leila V, Riviere M, Stieber D, Dreze P (2009). Contrasting epistatic interactions between rat quantitative trait loci controlling mammary cancer development. Mamm Genome.

[14] Moore JH (2003). The ubiquitous nature of epistasis in determining susceptibility to common human diseases. Hum Hered.

[15] Nagel RL (2005). Epistasis and the genetics of human diseases. C R Biol.

[16] Ambrosone CB, Freudenheim JL, Thompson PA, Bowman E, Vena JE (1999). Manganese superoxide dismutase (MnSOD) genetic polymorphism, dietary antioxidants, and risk of breast cancer. Cancer Res.

[17] Hursting SD, Lavigne JA, Berrigan D, Donehower LA, Davis BJ (2004). Diet-gene interactions in p53-deficient mice: Insulin-like Growth Factor-1 as a mechanistic target. J Nutr.

[18] McCullough ML, Stevens VL, Diver WR, Feigelson HS, Rodriguez C (2007). Vitamin D pathway gene polymorphism, diet, and risk of postmenopausal breast cancer: a nested case-control study. Breast Cancer Res.

[19] Hardman WE, Ion G, Akinsete JA, Witte TR (2011). Dietary walnut suppressed mammary gland tumorigenesis in the C(3)1 Tag mouse. Nutr Cancer.

[20] Gordon RR, Hunter KW, Sorensen P, Pomp D (2008). Genotype x diet interactions in mice predisposed to mammary cancer. I. Body weight and fat. Mamm Genome.

[21] Allan MF, Eisen EJ, Pomp D (2004). The M16 mouse: an outbred animal model of early onset polygenic obesity and diabesity. Obes Res.

[22] Allan MF, Eisen EJ, Pomp D (2005). Genomic mapping of direct and correlated responses to long-term selection for rapid growth rate in mice. Genetics.

[23] Guy CT, Cardiff RD, Muller WJ (1992). Induction of mammary tumors by expression of polyomavirus middle T oncogene: a transgenic mouse model for metastatic disease. Mol Cell Biol.

[24] Manly KF, Cudmore RH, Meer JM (2001). Map Manager QTX, cross-platform software for genetic mapping. Mamm Genome.

[25] Leamy LJ, Gordon RR, Pomp D (2011). Sex-, diet-, and cancer-dependent epistatic effects on complex traits in mice. Frontiers in Genet.

[26] Cheverud J, Wolf J, Brodie E, Wade M (2000). Detecting epistasis among quantitative trait loci. Epistasis and the Evolutionary Process.

[27] Li J, Ji L (2005). Adjusting multiple testing in multilocus analyses using the eigenvalues of a correlation matrix. Heredity.

[28] Lander E, Kruglyak L (1995). Genetic dissection of complex traits: guidelines for interpreting and reporting linkage results. Nat Genet.

[29] Wolf JB, Leamy LJ, Routman EJ, Cheverud JM (2005). Epistatic pleiotropy and the genetic architecture of covariation within early and late-developing skull trait complexes in mice. Genetics.

[30] Wendell DL, Herman A, Gorski J (1996). Genetic separation of tumor growth and hermorrhagic phenotypes in an estrogen-induced tumor. Proc Nat Acad Sci.

[31] Hunter KW, Broman KW, Le Voyer T, Lukes L, Cozma D (2001). Predisposition to efficient mammary tumor metastatic progression is linked to the breast cancer metastasis suppressor gene *Brms1*. Cancer Res.

[32] Gordon RR, La Merrill M, Hunter KW, Sorensen P, Threadgill DW (2010). Dietary fat-dependent transcriptional architecture and copy number alterations associated with modifiers of mammary cancer metastasis. Clin Exper Metast.

[33] La Merrill M, Gordon RR, Hunter KW, Threadgill DW, Pomp D (2010). Dietary fat alters pulmonary metastasis of mammary cancers through cancer autonomous and non-autonomous changes in gene expression. Clin Exper Metast.

[34] Oshima RG, Lesperance J, Munoz V, Hebbard L, Ranscht B (2004). Angiogenic acceleration of Neu induced mammary tumor progression and metastasis. Cancer Res.

